# Outcomes of women with congenital heart disease admitted to acute-care hospitals for delivery in Japan: a retrospective cohort study using nationwide Japanese diagnosis procedure combination database

**DOI:** 10.1186/s12872-021-02222-z

**Published:** 2021-08-27

**Authors:** Manabu Nitta, Sayuri Shimizu, Makoto Kaneko, Kiyohide Fushimi, Shinichiro Ueda

**Affiliations:** 1 Department of Health Data Science, Graduate School of Data Science, Yokohama City University, 22-2 Seto, Kanazawa, Yokohama, 236-0027 Japan; 2 Department of Medical Science and Cardiorenal Medicine, Yokohama City University Graduate School of Medicine, 3-9 Fukuura, Kanazawa, Yokohama, 236-0004 Japan; 3 Department of Health Policy and Informatics, Tokyo Medical and Dental University Graduate School of Medicine, 1-5-45 Yushima, Bunkyo-ku, Tokyo, 1138519 Japan

**Keywords:** Adult congenital heart disease, Pregnancy, Nationwide survey, Diagnosis procedure combination database

## Abstract

**Background:**

The number of women with congenital heart disease (CHD) who are of childbearing age is increasing due to advancements in medical management. Nonetheless, data on the outcomes of delivery in women with CHD remain limited. Therefore, we conducted a retrospective cohort study using a nationwide database of deliveries by women with CHD.

**Methods:**

Deliveries by women with CHD discharged from acute-care hospitals between April 2017 and March 2018 were identified based on the Diagnosis Procedure Combination database which covers almost all acute-care hospitals in Japan. By using this database, we tried to include relatively high-risk deliveries by women with CHD. Subjects were divided into three groups according to the underlying disease complexity: simple, moderate, and great complexity. The clinical characteristics and incidence of peripartum cardiovascular events were compared among the three groups.

**Results:**

A total of 249 deliveries from 107 hospitals were included. The largest facility had 29 deliveries per year. Given the uncertainty of underlying cardiac anomalies, 48 women were excluded, and the remaining 201 women (median age, 32 years) were analyzed. In-hospital maternal death, use of extracorporeal membrane oxygenation, intra-aortic balloon pump, pacemaker, and direct current cardioversion were not observed. Nine patients (4.5%) required intravenous diuretic administration. However, the difference in the frequency of diuretic use was not significant among the three groups (simple, 1.9%; moderate, 7.2%; great, 6.9%; *P* = 0.204). One participant required valve replacement surgery at 22 days after a successful cesarean section. As the disease complexity increased, deliveries occurred more frequently at university hospitals (simple, 41.7%; moderate, 52.2%; great, 72.4%; *P* = 0.013) and the length of hospitalization was significantly longer, with median durations of 9.0 (interquartile range [IQR] 7.0–11.0) days, 10.0 (IQR 8.0–24.0) days, and 11.0 (IQR 8.0–36.0) days in the simple, moderate, and great complexity groups, respectively (*P* = 0.002).

**Conclusions:**

Appropriate patient selection and management by specialized tertiary institutions may contribute to positive outcomes in pregnancies in women with CHD.

**Supplementary Information:**

The online version contains supplementary material available at 10.1186/s12872-021-02222-z.

## Introduction

The number of women with congenital heart disease (CHD) who are of childbearing age and wish to become pregnant is increasing due to advancements in cardiac surgery and medical management during childhood [1–3]. Majority of women with CHD are concerned about their ability to become pregnant and deliver [4].

Pregnancy can pose a risk to both mothers and their offspring, particularly among women with CHD, through residual lesions and sequelae after corrected or uncorrected underlying cardiac anomalies [5]. Pregnancy causes a circulatory burden primarily due to the increased circulating plasma volume, which can have a considerable impact even on healthy women [6, 7]. In several clinical guidelines, painless vaginal delivery with epidural analgesia is recommended depending on disease severity, as it can decrease the cardiac load during delivery by reducing labor pain and stress [8–10].

The incidence and outcomes of pregnancy and childbirth in women with CHD must be thoroughly investigated in order to develop medical strategies and provide improved medical care for those who wish to become pregnant. The incidence of childbirth in pregnant women with CHD has not yet been reported. The Japanese guidelines estimated that childbirth by women with cardiac diseases accounts for 0.5–1.0% of the total pregnancies in Japan [9]. Nonetheless, the data are scant due to the relatively small number of patients and institutes included as well as the exclusion of critically ill patients [11–15]. Additionally, the definitions and severities of peripartum cardiovascular events have been inconsistent among studies [5, 14, 15].

Herein, we conducted a retrospective cohort study based on the nationwide database of acute-care institutions in Japan to comprehensively explore the outcomes of delivery in women with CHD who were admitted to acute-care hospitals.

## Methods

### Study design

This is a retrospective cohort study based on a nationwide database of acute-care institutions in Japan.

### Data source

Data from the Diagnosis Procedure Combination (DPC) database were utilized. The DPC database covers 1664 acute-care hospitals, accounting for 54.1% of all hospital beds in Japan in April 2017 [16, 17], and is highly validated, particularly for primary diagnoses and procedure records [18]. Our DPC database-based study included 1253 out of 1664 (75.3%) acute-care hospitals. It is reasonable to utilize the nationwide database of acute-care institutions to survey deliveries by women with CHD because, in Japan, normal deliveries are not covered by insurance and therefore not covered by the medical insurance database. However, patients requiring medical interventions are likely to be admitted to acute-care hospitals and, therefore, covered by insurance and medical insurance databases. Thus, we tried to include relatively high-risk deliveries by women with CHD. The database includes the following information for each patient: unique hospital identifier, date of admission, age and sex, primary diagnoses and comorbidities, consciousness on arrival and discharge defined by Japan Coma Scale [19], purpose of the admission (examination, scheduled treatment, or emergency treatment), treatments and devices, diagnostic and therapeutic procedures, hospitalization stay, total medical cost [20], and discharge status, including in-hospital death, functional status at discharge according to the Barthel index, and discharge locations [17]. There are six categories of diagnoses, each with a limited number of recordable diseases [18]. One diagnosis each is coded for “main diagnosis”, “admission-precipitating diagnosis”, “the most resource consuming diagnosis”, and “second resource consuming diagnosis”. A maximum of four diagnoses each can be coded for “comorbidities present at time of admission” and “conditions arising after admission”.

### Study population

Data on childbirth by women with CHD who were discharged between April 1, 2017, and March 31, 2018, were obtained. The last admission was counted for patients with multiple admissions. First, women with CHD who were hospitalized during the study period were designated based on the International Classification of Diseases (ICD)-10 diagnosis codes (Additional file 1: Table S1). Parturition was also identified based on the K-codes indicating delivery (Additional file 2: Table S2). When extracting participants, delivery was identified by diagnosis coded for “main diagnosis”, “admission-precipitating diagnosis”, or “the most resource consuming diagnosis”. In addition, CHD was identified by diagnoses coded for “comorbidities present at time of admission”. We classified the subjects according to the complexity of CHD according to the American College of Cardiology (ACC) and the 2008 American Heart Association (AHA) guidelines [10], and assigned them one of the following three groups: simple, moderate, and great complexity. When several diagnoses were logged in one woman, the woman was classified according to the most important diagnosis for determining disease complexity. For example, both ventricular septal defect (VSD) and coarctation of the aorta (CoA) were logged in 1 woman, VSD is usually classified as simple complexity, and CoA as moderate. Therefore, the woman was classified as moderate complexity. For analyses, we excluded cases with uncertain underlying cardiac anomalies who were coded as Q249 according to the ICD-10 code, which suggests “congenital malformation of heart, unspecified”.

### Variables

The patients’ background data including age, sex, height, weight, underlying cardiac anomalies, smoking history, use of ambulance on admission, incidence of emergency hospitalization, type of hospital (university hospital or other), and distance between home and hospital were obtained. In Japan, there are a limited number of hospitals that can accommodate the delivery of women with CHD [21]. Furthermore, fewer hospitals can manage delivery of women with high-complexity CHD (i.e., more severe women). Therefore, in Japan, women with high-complexity CHD have to deliver at specialized facilities a little away from their home. Obstetric data including weeks of gestation (both on admission and at delivery), delivery methods (vaginal delivery including instrumental delivery, emergent cesarean section, or elective cesarean section), and volume of blood loss during delivery, were also identified. Data regarding anesthesia were collected to identify painless delivery or whether cesarean section was performed under general anesthesia or spinal anesthesia.

### Outcome measures

In-hospital outcomes included death, heart failure, circulatory insufficiency, arrhythmic events, utilization of intensive care unit (ICU), and length of hospitalization. The codes of diseases suggesting heart failure, circulatory insufficiency, and arrhythmic events could not be extracted and identified due to the complexity and inaccuracy of the extraction. Instead, we extracted the peripartum use of mechanical support and management, including extracorporeal membrane oxygenation (ECMO), intra-aortic balloon pump (IABP), pacemaker for symptomatic bradycardia, and direct current cardioversion for discontinuation of tachyarrhythmia. We also extracted the peripartum intravenous administrations of inotropes, diuretics, and antiarrhythmic agents. Thus, we identified the relatively severe conditions in pregnant and parturient women with CHD. We defined exacerbation of heart failure as intravenous administration of diuretics. We also identified arrhythmic event as arrhythmia requiring cardioversion, pacemaker, or intravenous administration of antiarrhythmic agents. In addition, we also detected the administration of antibiotic agents, heparin, and oral anticoagulants, including warfarin and direct oral anticoagulants (DOACs).

### Statistical analysis

Nominal-level data were expressed as percentages, whereas medians with interquartile ranges were calculated for continuous variables. The subjects’ characteristics were then compared among the three groups using the χ^2^ and Kruskal–Wallis tests for nominal and continuous variables, respectively. All statistical tests were two-sided, with a 5% level of significance. Statistical analyses were performed using RStudio version 4.0.0 (RStudio, Boston, MA, USA).

### Ethics

This study was approved by the ethics committee of Tokyo Medical and Dental University (approval number: M2000-78-16) and was conducted in accordance with ethical standards as described in the 2002 Declaration of Helsinki. Informed consent was waived due to the anonymous nature of the data.

## Results

### Characteristics of women with CHD included in the study

**Fig. 1:**
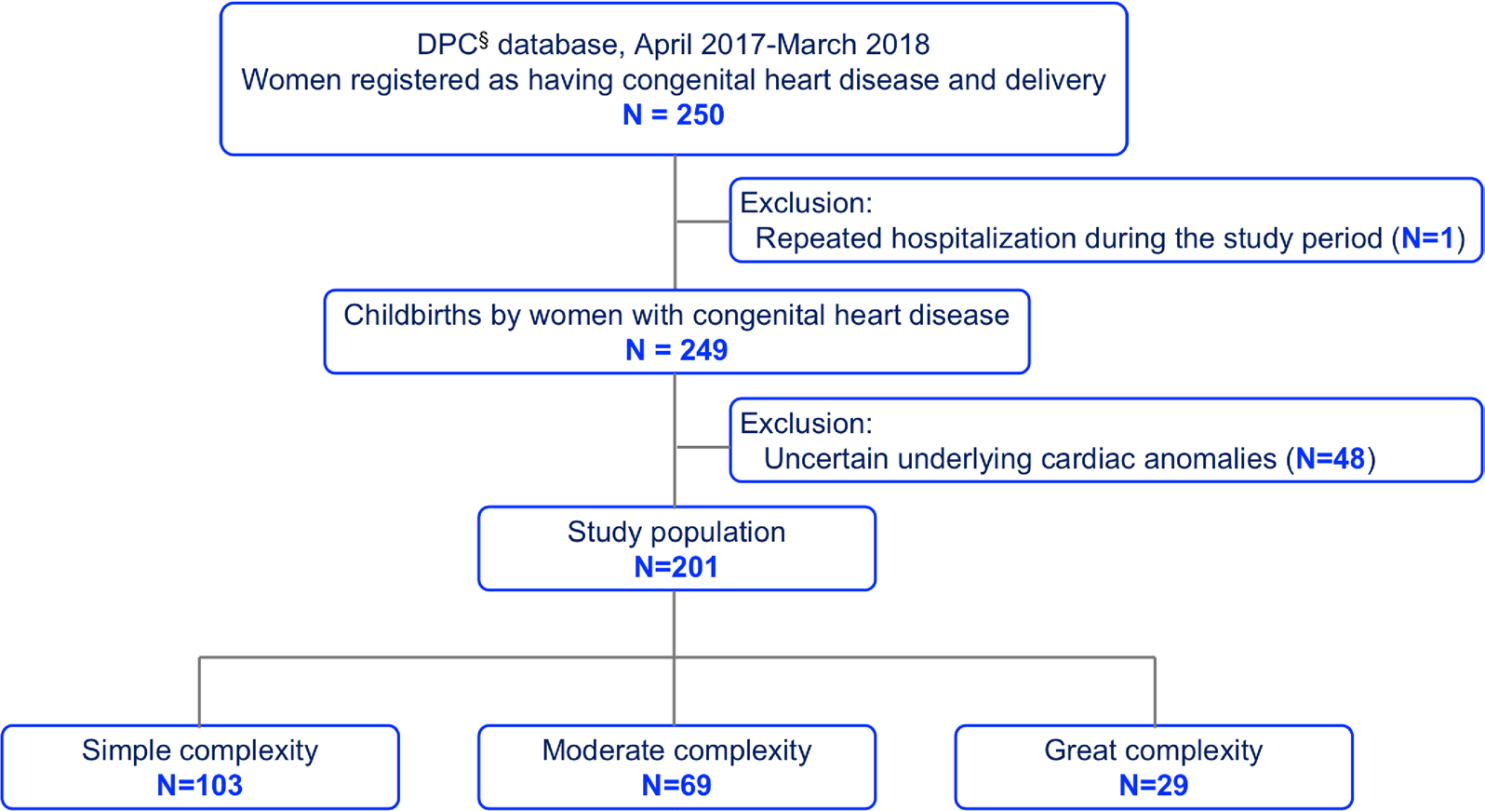
Flow diagram of the study population. § DPC, diagnosis procedure combination

A total of 250 pregnancies and delivery-related hospitalizations in women with CHD were identified between April 1, 2017, and March 31, 2018. One woman was hospitalized twice during the study period: once for cervical cerclage aimed at reducing the risk of early birth and once for delivery. Table 1 summarizes the characteristics of all 249 women with CHD who gave birth and the frequency of missing data. Cesarean sections were performed in 73% of the subjects, and 55% of all deliveries occurred at university hospitals. Data regarding the distance between home and hospital were the ones missing most frequently, equivalent to 31.3%. Two women with pulmonary arterial hypertension (PAH) were identified; this is PAH associated with an atrial septal defect and the other with total anomalous pulmonary venous connection. Figure 1 illustrates the flow diagram of the study population. Forty-eight women coded as Q249 of ICD-10 suggesting “congenital malformation of heart, unspecified” were excluded from the analyses. Table 2 shows the details of the types of disease according to disease complexity. As underlying cardiac anomalies, VSD, tetralogy of Fallot, and univentricular heart were most frequently observed in the simple, moderate, and great complexity groups, respectively. The clinical differences among the three groups are shown in Table 3. As the disease complexity increased, the gestational weeks of hospitalization and delivery were significantly earlier. In addition, as the disease complexity increased, the distance between the home and hospitals increased, and deliveries were performed more frequently at university hospitals. However, regarding the distance between home and hospitals, missing data were more frequently observed in the great complexity group than in the simple and moderate groups (simple: 21.4%, moderate: 33.3%, great: 55.2%).

**Table 1:** Clinical characteristics of the pregnant women with congenital heart disease

Total N = 249	n (%) or median (inter-quartile range)	Number of missing data
Age (years)	32 (28–35)	0 (0.0%)
Height (cm)	157 (153–160)	4 (1.6%)
Weight (kg)	58.2 (52.4–64.4)	2 (0.8%)
Past or current smokers	18 (7.2%)	18 (7.2%)
Ambulance	9 (3.6%)	0 (0.0%)
Emergent hospitalization	25 (10.0%)	0 (0.0%)
Referral from other hospitals	198 (79.5%)	0 (0.0%)
Weeks of gestation		
On admission (weeks)	37 (35–38)	9 (3.6%)
At delivery (weeks)	37 (36–38)	9 (3.6%)
< 22 weeks	5 (2.0%)	
22–37 weeks	70 (28.1%)	
37 ≤ weeks	165 (66.3%)	
Delivery methods		0 (0.0%)
Vaginal	68 (27.3%)	
Vacuum extraction delivery	40 of 68 (58.9%)	
Forceps delivery	0 of 68 (0.0%)	
Cesarean section (emergent)	72 (28.9%)	
Cesarean section (elective)	109 (43.8%)	
Volume of intrapartum hemorrhage (mL)	606 (390–872)	12 (4.8%)
Utilization of intensive care unit	75 (30.1%)	0 (0.0%)
Length of stay (days)	9 (8–17)	0 (0.0%)
Distance between home and hospital (km)	9.8 (5.2–18.0)	78 (31.3%)
Types of hospitals		0 (0.0%)
University hospitals	137 (55.0%)	
Other hospitals	112 (45.0%)

**Table 2:** Types of diseases according to disease complexity

Degree of complexity	Types of diseases	Number of cases (total N = 249)
Simple		103 (41.4%)
	VSD	67
	ASD	26
	PDA	3
	AS^a^	1
	AR	5
	MR	1
Moderate		69 (27.7%)
	TOF	31
	CoA	8
	PS	7
	AVSD	6
	Ebstein’s anomaly	3
	AS^b^	3
	PR	2
	TS	2
	TAPVC	1
	Others	6
Great		29 (11.6%)
	UVH	11
	d-TGA	3
	ccTGA	4
	PA	5
	DORV	6
Uncertain		48 (19.3%)

AR, aortic regurgitation; AS, aortic stenosis; ASD, atrial septal defect; AVSD, atrioventricular septal defect; ccTGA, congenitally corrected transposition of the great arteries; CoA, coarctation of the aorta; d-TGA, dextro-transposition of the great arteries; DORV, double outlet right ventricle; MR, mitral regurgitation; PA, pulmonary atresia; PDA, patent ductus arteriosus; PR, pulmonary regurgitation; PS, pulmonary stenosis; TAPVC, total anomalous pulmonary venous connection; TOF, tetralogy of Fallot; TS, tricuspid stenosis; UVH, univentricular heart; VSD, ventricular septal defect

^a^Isolated congenital aortic valve disease (stenosis)

^b^Subvalvular aortic stenosis or supravalvular aortic stenosis

**Table 3:** Differences in clinical characteristics according to disease complexities

Total N= 201	n (%) or median (inter-quartile range)			
	Simple n = 103	Moderate n = 69	Great n = 29	*P* value
Age (years)	32 (28–35)	32 (28–35)	32 (28–34)	0.825
Height (cm)	156 (153–160)	157 (151–161)	157 (154–160)	0.642
Weight (kg)	58.9 (52.2–64.9)	57.0 (52.3–62.4)	58.1 (51.2–63.5)	0.645
Past or current smokers	11 (10.7%)	4 (5.8%)	1 (3.4%)	0.291
Ambulance	4 (3.9%)	2 (2.9%)	0 (0.0%)	0.554
Emergent hospitalization	9 (8.7%)	8 (11.6%)	3 (10.3%)	0.826
Referral from other hospital	79 (76.7%)	53 (76.8%)	22 (75.9%)	0.994
Weeks of gestation				
On admission (weeks)	38 (36–39)	37 (33–38)	36 (34–37)	0.004
At delivery (weeks)	38 (37–39)	37 (35–38)	37(36–38)	0.028
Delivery Methods				0.748
Vaginal	33 (32.0%)	20 (29.0%)	8 (27.6%)	
Cesarean section (emergent)	29 (28.2%)	16 (23.2%)	6 (20.7%)	
Cesarean section (elective)	41 (39.8%)	33 (47.8%)	15 (51.7%)	
Distance between home and hospital (km)	8.6 (4.3–13.5)	10.0 (6.4–21.8)	14.9 (12.3–19.9)	0.004
Types of hospitals				0.013
University hospitals	43 (41.7%)	36 (52.2%)	21 (72.4%)	
Other hospitals	60 (58.3%)	33 (47.8%)	8 (27.6%)	

### Delivery and anesthetic methods

**Fig. 2:**
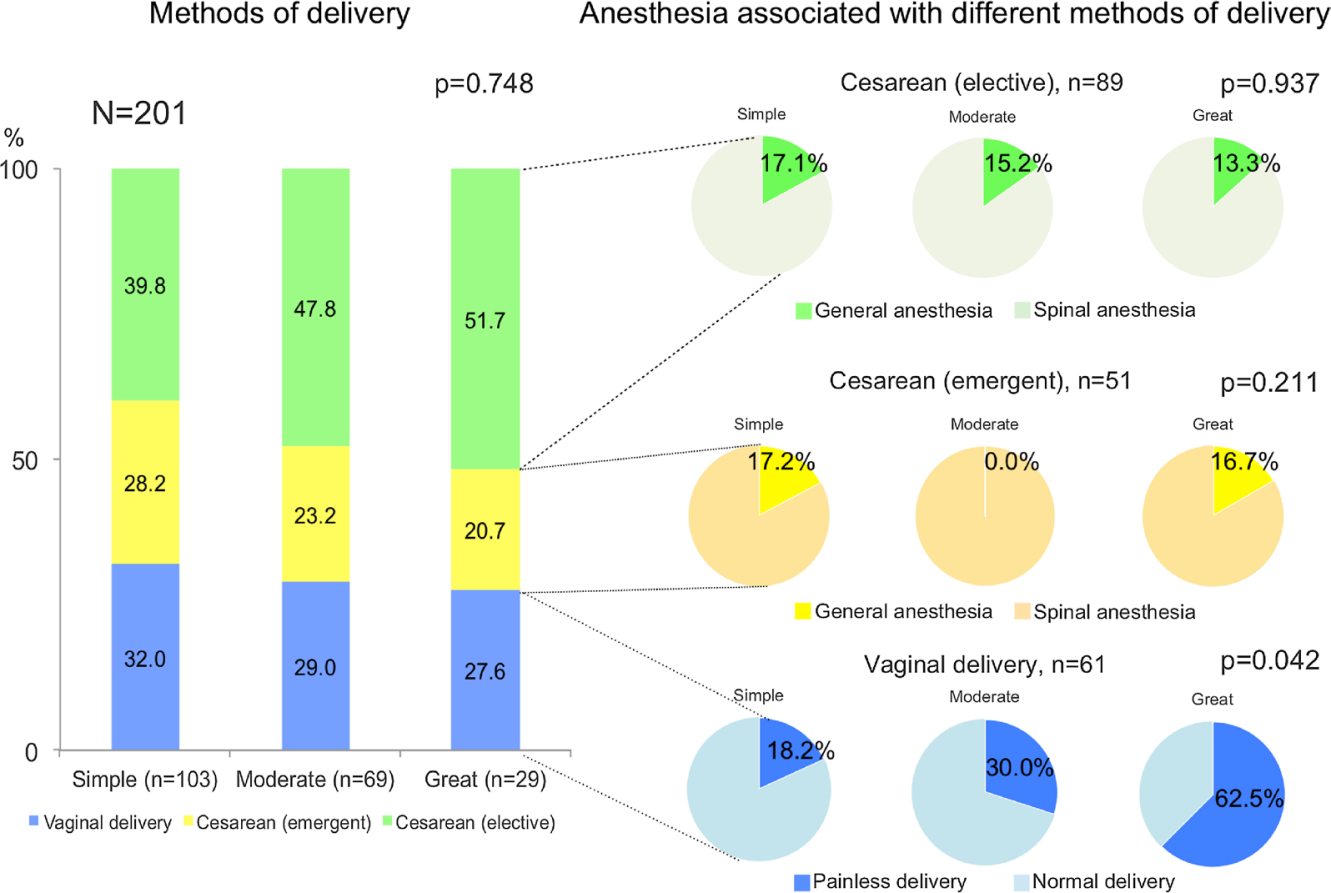
Delivery and anesthetic methods according to disease complexity

The delivery methods were analogous among the three groups (Table 3, Fig. 2). Regarding vaginal delivery, painless delivery using epidural or spinal analgesia was more frequently observed as the disease complexity increased, which was statistically significant. Regarding cesarean section, the use of general anesthesia was similar among the three groups.

### In-hospital outcomes

In-hospital death was not observed in any group or the 48 excluded subjects (Table 4). None of the patients required ECMO, IABP, cardioversion, or pacemaker. As disease complexity increased, the length of hospitalization was significantly increased. On the other hand, the peripartum exacerbation of heart failure requiring intravenous diuretics, arrhythmic events requiring intravenous antiarrhythmic agents, use of antibiotic agents, and use of anticoagulants except for heparin use before delivery did not differ among the three groups. No DOACs were administered. ICU utilization significantly differed among the three groups. However, requirement of staying in the ICU did not follow the order of disease complexity (i.e., ICU stay was most frequently required in the moderate complexity group).

**Table 4:** In-hospital outcomes and readmission to the same hospital within 1 year after delivery

Total N = 201	n (%) or median (inter-quartile range)			
	Simple n = 103	Moderate n = 69	Great n = 29	*P* value
In-hospital outcome				
Use of ECMO^a^, IABP^b^	0 (0.0%)	0 (0.0%)	0 (0.0%)	
In-hospital death	0 (0.0%)	0 (0.0%)	0 (0.0%)	
Cardiac surgery during hospitalization	1 (1.0%)	0 (0.0%)	0 (0.0%)	0.620
Volume of intrapartum hemorrhage (mL)	598 (392–874)	569 (372–800)	660 (368–809)	0.686
Utilization of intensive care unit	22 (21.4%)	29 (42.0%)	7 (24.1%)	0.011
Length of hospitalization (days)	9.0 (7.0–11.0)	10.0 (8.0–24.0)	11.0 (8.0–36.0)	0.002
Exacerbation of heart failure (requiring intravenous diuretics)	2 (1.9%)	5 (7.2%)	2 (6.9%)	0.204
Arrhythmic event				
requiring cardioversion or pacemaker	0 (0.0%)	0 (0.0%)	0 (0.0%)	
requiring intravenous antiarrhythmics	1 (1.0%)	0 (0.0%)	0 (0.0%)	0.620
Peripartum intravenous use of antibiotic agents	81 (78.6%)	55 (78.3%)	22 (75.9%)	0.950
Use of heparin				
Before delivery	0 (0.0%)	1 (1.4%)	2 (6.9%)	0.026
After delivery	3 (2.9%)	2 (2.9%)	1 (3.4%)	0.988
Use of warfarin after delivery	2 (1.9%)	1 (1.4%)	1 (3.4%)	0.810

^a^ECMO, extracorporeal membrane oxygenation

^b^IABP, intra-aortic balloon pump

One participant with a ventricular septal defect underwent emergency hospital admission because of active infective endocarditis (IE) at 28 weeks gestation and had valve replacement surgery performed after successful delivery by cesarean section at 32 weeks gestation.

## Discussion

To the best of our knowledge, our study is the first to evaluate the nationwide outcomes of delivery in women with CHD in Japan. The most important findings in our study are as follows: first, the number of childbirths in women with CHD who required medical interventions and were admitted to acute-care hospitals was 249 annually in Japan. Notably, among those, approximately 100 childbirths were in women classified as having moderate to great complexity. Second, delivery in selected women with CHD were considerably safe, and the outcomes were acceptable. Considering that more than half of the subjects delivered at university hospitals, the current study highlights the importance of specialized facilities that have multidisciplinary teams capable of managing the pregnancies and deliveries of women with CHD. Our study may encourage women with CHD who are anxious and perhaps pessimistic about their pregnancy and delivery. It should be important that women with CHD estimated to be at high peripartum risk are referred to specialized facilities with multidisciplinary teams from the stage of pre-pregnancy counseling.

### Peripartum adverse cardiovascular events and readmissions within 1 year

During the study period, no deaths associated with childbirth were observed. This was consistent with previous reports from Japan [13–15]. On the other hand, the incidence of peripartum cardiovascular events, most of which were heart failure, arrhythmias, and thromboembolic events, were discordant between the studies [5, 13–15]. The discordance may potentially be attributed to the definition or severity of adverse events. A previous literature review, which included 48 articles describing a total of 2491 pregnancies, demonstrated that significant cardiac complications were observed in 11% of the pregnancies in women with CHD [5]. In this literature review, cardiac complications were defined as episodes “*requiring treatment*”; therefore, relatively mild cases may have been included. On the other hand, we attempted to identify more severe conditions. In this DPC database-based study, the identification of cardiovascular diseases during the peripartum period might be inaccurate. Therefore, we attempted to identify the codes indicating treatments including the peripartum use of ECMO, IABP, pacemaker, direct current cardioversion, and intravenous drugs. Oral administration was only allowed for anticoagulation agents, including warfarin and DOACs. Thus, we could identify and extract critically ill episodes. According to this definition, the incidence of adverse cardiovascular events around delivery was low, even in women with moderate to great CHD complexity. It could be considered that pregnant women with CHD were appropriately selected and referred to the expert facilities and received optimal risk stratification and management by the pregnancy heart team provided.

Overall, intravenous antibiotics were administered to 189 women (75.9%) as IE prophylaxis or for obstetric reasons. One pregnant woman with active IE was admitted to the hospital emergency room and successfully delivered via cesarean section; she subsequently underwent valve replacement surgery. IE is rare, with an overall estimated annual incidence of 1 per 1000 in patients with CHD [22, 23] and 3–12 per 1000 in patients with prosthetic valves [24]. The incidence of bacteremia after vaginal delivery is 0–5% [25, 26]. In general, the indication for prophylaxis applies to non-pregnant patients, and routine antibiotic administration is not recommended during vaginal or cesarean delivery because of the lack of convincing evidence [8, 10]. On the other hand, the Japanese guidelines recommend the prophylactic administration of antibiotic agents at the time of delivery in the high-risk IE group for the following reasons [8, 27–29]: (1) severe infections may occur in the moderate- to high-risk groups; (2) the cost of antibiotic agents is relatively low; and (3) allergic reactions to antibiotic agents, which are considered risk factors, are not frequently observed. However, further discussion is warranted to elucidate the aforementioned point.

The length of hospitalization significantly increased with increased disease complexity. However, the ICU stay did not follow the order of disease complexity. Approximately 30% of subjects were required to stay in the ICU, the rate of which was considerably higher than that previously reported [30]. Such variation mainly reflects the differences in the indication for ICU utilization or in the cultural context [31, 32]. In Japan, routine admission of high-risk pregnant women to the maternal–fetal ICU is recommended.

A previous retrospective cohort study using DPC database compared clinical features and peripartum outcomes between pregnant women with cardiac diseases and those without cardiac diseases, in which, 302 deliveries by women with CHD were identified for 6 years [13]. However, the identification of CHD was not exhaustive, therefore, types and numbers of CHD included were relatively small. On the other hand, we comprehensively identified women with CHD. Moreover, the clinical characteristics and incidence of peripartum cardiovascular events were investigated according to the disease complexity.

### Emerging requirement of the pregnancy heart team

Specialized facilities may play an important role in the achievement of the peripartum mortality of zero and the low incidence of adverse cardiovascular events in women with CHD admitted for delivery. In our DPC database-based study, hospitals other than university hospitals are also CHD-specialized facilities with capacity comparable to university hospitals. The DPC database covers almost all acute-care hospitals in Japan. In general, acute-care hospitals suggest high-volume institutes in Japan. We consider that outcome of deliver by women with CHD may not differ between in university hospitals and in other acute-care hospitals, however, the difference of impact on delivery outcomes between university hospitals and other hospitals requires further study.

These expert centers should have a multidisciplinary team, termed “pregnancy heart team” [8]. This team would include adult CHD cardiologists, pediatric cardiologists, obstetricians, anesthetists, and other healthcare providers who are familiar with the management of high-risk pregnancies in women with cardiac diseases. The team should provide pre-pregnancy counseling, risk stratification of obstetric and offspring complications, and management during pregnancy and delivery. However, the term “pregnancy heart team” is not yet common or widely recognized in Japan. As childbirths in women with CHD would be expected to increase with the increasing number of adult CHD patients [14, 15], the importance of the pregnancy heart team will therefore increase in the future. It should be recognized that women with CHD estimated to be at high peripartum risk need to be referred to specialized facilities with multidisciplinary teams from the stage of pre-pregnancy counseling.

### Limitations

Although, the DPC database included a larger number of childbirths than previous Japanese cohort studies [11–15], which enabled us to evaluate the current nationwide status, our study had several limitations. First, some data could not be identified from the DPC database. In fact, we could not identify patients with cyanosis or aortopathy. The DPC system does not also include healthy childbirths and those of women with minor heart diseases; therefore, these could not be evaluated. Most mild cardiac diseases include simple complexities with preserved ventricular and valvular function and without significant residual lesions and sequelae, including severe PAH. Our study focused on childbirths in women with significant CHD who required DPC coverage. On the other hand, also we might not enroll women estimated high peripartum risk, because they would have been recommended contraception, which might cause selection bias. Including only women surviving to delivery was another selection bias which affected the low (0%) mortality. Furthermore, data on outcomes might be missing. In fact, regarding instrumental delivery, vacuum deliveries could be identified, however, forceps deliveries could not. Second, we could not detect data regarding obstetric and neonatal complications. However, obstetric complications, including pregnancy-induced hypertension, pre-eclampsia, eclampsia, and hemolysis, elevated liver enzymes, and low platelet count (HELLP) syndrome, have been reported to be less prevalent than peripartum cardiac complications [5]. The incidence of low birth weight, preterm birth, and offspring mortality increases with increasing disease complexity and severity [5, 14, 15]. Combining the maternal and neonatal data was challenging; when we confirmed, data on birth weight had not been correctly recorded. Third, as generally applied to database-based studies, CHD severity was undetermined. In most cases, disease complexity would be proportional to disease severity, however, occasionally would not (e.g. atrial septal defect with PAH). Several risk stratification systems have been proposed to evaluate pregnant women with CHD, including the cardiac disease in pregnancy (CARPREG) risk score [33], modified World Health Organization (WHO) classification [34], and Zwangerschap bij vrouwen met een Aangeboren HARtAfwijking-II, translated as Pregnancy in women with CHD II risk index (ZAHARA) score [35]. These stratification systems require more precise and complicated medical records including WHO functional class, morphology of systemic ventricle, systemic ventricular function, Fontan circulation, degree of cyanosis, valvular diseases, mechanical heart valve, or pulmonary hypertension, which are not included in the DPC database. Therefore, we could not calculate and compare to the established maternal mortality scores for CHD. Only 2 women with PAH were identified, however, severity of PAH could not be determined. Therefore, we classified CHD according to the complexity of CHD defined by ACC/AHA guidelines 2008 [10], which could be easily classified using only CHD diagnoses and are generally proportional to disease severity. Fourth, we could not assess miscarriage, abortion, counselled against pregnancy, and maternal adverse events prior to admission for delivery, which are important factors when discussing pregnancies and deliveries in women with CHD.

### Perspectives to improve childbirths by women with CHD

Delivery outcomes are acceptable for women with CHD who are appropriately selected and managed by specialized facilities. Generally, women with CHD are worried and pessimistic about their ability to successfully conceive and deliver [4]. As treatments and management of CHD during childhood continue to improve, more patients with CHD can enter adulthood in a better condition and a larger number of women with complicated CHD can conceive and deliver safely. We emphasize the importance of evaluation by the pregnancy heart team from pre-pregnancy counseling to postpartum management.

In addition to the status of maternal adverse events throughout pregnancy and neonatal adverse events, those regarding miscarriage, abortion, and counselled against pregnancy due to maternal CHD remain unclear. Therefore, a more comprehensive registry-based survey is required.

## Conclusions

Appropriate patient selection and management were provided in pregnancies in women with CHD by specialized facilities with multidisciplinary teams in Japan. With the increasing number of adult CHD patients, the role and importance of the pregnancy heart team will increase in the future. Furthermore, the status of miscarriage, abortion, and counselled against pregnancy due to maternal CHD warrants further elucidation.

## Supplementary Information

**Additional file 1. Table S1:** International Classification of Diseases (ICD)-10 diagnosis codes identifying congenital heart diseases.

**Additional file 2. Table S2:** K-codes indicating delivery.

## Data Availability

The datasets used and/or analyzed during the current study are available from the corresponding author on reasonable request.

## Abbreviations

CHD: Congenital heart disease
DPC: Diagnosis procedure combination
ICD: International classification of diseases
ICU: Intensive care unit
ECMO: Extracorporeal membrane oxygenation
IABP: Intra-aortic balloon pump
DOACs: Direct oral anticoagulants
IE: Infective endocarditis
HELLP: Hemolysis elevated liver enzymes and low platelet count
CARPREG: Cardiac disease in pregnancy
WHO: World health organization
ZAHARA: Zwangerschap bij Aangeboren HARtAfwijkingen
ACC: American college of cardiology
AHA: American heart association
