# Supplementary material for: Outcomes of women with congenital heart disease admitted to acute-care hospitals for delivery in Japan: a retrospective cohort study using nationwide Japanese diagnosis procedure combination database
Source: BMC Cardiovasc Disord. 2021 Aug 27;21:409. doi: 10.1186/s12872-021-02222-z (PMC8393443; doi:10.1186/s12872-021-02222-z)
Supplement: Supplementary file 1 — Additional file 1. Table S1: International Classification of Diseases (ICD)-10 diagnosis codes identifying congenital heart diseases. [file 12872_2021_2222_MOESM1_ESM.docx]

**SUPPLEMENTAL MATERIAL**

These tables are intended for publication as an online data supplement.

**Supplementary Table S1.**

International Classification of Diseases (ICD)-10 diagnosis codes identifying congenital heart diseases

| Code | diagnosis 1 | diagnosis 2 |
| --- | --- | --- |
| Q200 | Common arterial trunk | Persistent truncus arteriosus |
| Q201 | Double outlet right ventricle |  |
| Q202 | Double outlet left ventricle |  |
| Q203 | Discordant ventriculoarterial connection | Transposition of great vessels (complete) |
| Q204 | Double inlet ventricle | Single ventricle |
| Q205 | Discordant atrioventricular connection | Corrected transposition |
| Q206 | Isomerism of atrial appendages | Isomerism of atrial appendages with asplenia or polysplenia |
| Q208 | Other congenital malformations of cardiac chambers and connections |  |
| Q209 | Congenital malformation of cardiac chambers and connections, unspecified |  |
| Q210 | Ventricular septal defect |  |
| Q211 | Atrial septal defect |  |
| Q212 | Atrioventricular septal defect |  |
| Q213 | Tetralogy of Fallot |  |
| Q214 | Aortopulmonary septal defect |  |
| Q218 | Other congenital malformations of cardiac septa | Eisenmenger defect, Pentalogy of Fallot |
| Q219 | Congenital malformation of cardiac septum, unspecified |  |
| Q220 | Pulmonary valve atresia |  |
| Q221 | Congenital pulmonary valve stenosis |  |
| Q222 | Congenital pulmonary valve insufficiency |  |
| Q223 | Other congenital malformations of pulmonary valve | Congenital malformation of pulmonary valve NOS |
| Q224 | Congenital tricuspid stenosis | Tricuspid atresia |
| Q225 | Ebstein anomaly |  |
| Q226 | Hypoplastic right heart syndrome |  |
| Q228 | Other congenital malformations of tricuspid valve |  |
| Q229 | Congenital malformation of tricuspid valve, unspecified |  |
| Q230 | Congenital stenosis of aortic valve |  |
| Q231 | Congenital insufficiency of aortic valve | Bicuspid aortic valve |
| Q232 | Congenital mitral stenosis |  |
| Q233 | Congenital mitral insufficiency |  |
| Q234 | Hypoplastic left heart syndrome |  |
| Q238 | Other congenital malformations of aortic and mitral valves |  |
| Q239 | Congenital malformation of aortic and mitral valves, unspecified |  |
| Q240 | Dextrocardia |  |
| Q241 | Levocardia |  |
| Q242 | Cor triatriatum |  |
| Q243 | Pulmonary infundibular stenosis |  |
| Q244 | Congenital subaortic stenosis |  |
| Q245 | Malformation of coronary vessels |  |
| Q246 | Congenital heart block |  |
| Q248 | Other specified congenital malformations of heart |  |
| Q249 | Congenital malformation of heart, unspecified |  |
| Q250 | Patent ductus arteriosus |  |
| Q251 | Coarctation of aorta | Interuption of aorta |
| Q252 | Atresia of aorta |  |
| Q253 | Stenosis of aorta | Supravalvular aortic stenosis |
| Q254 | Other congenital malformations of aorta |  |
| Q255 | Atresia of pulmonary artery |  |
| Q256 | Stenosis of pulmonary artery |  |
| Q257 | Other congenital malformations of pulmonary artery |  |
| Q258 | Other congenital malformations of great arteries |  |
| Q259 | Congenital malformation of great arteries, unspecified |  |
| Q260 | Congenital stenosis of vena cava |  |
| Q261 | Persistent left superior vena cava |  |
| Q262 | Total anomalous pulmonary venous connection |  |
| Q263 | Partial anomalous pulmonary venous connection |  |
| Q264 | Anomalous pulmonary venous connection, unspecified |  |
| Q268 | Other congenital malformations of great veins |  |
| Q269 | Congenital malformation of great vein, unspecified |  |
