# Supplementary material for: Outcomes of women with congenital heart disease admitted to acute-care hospitals for delivery in Japan: a retrospective cohort study using nationwide Japanese diagnosis procedure combination database
Source: BMC Cardiovasc Disord. 2021 Aug 27;21:409. doi: 10.1186/s12872-021-02222-z (PMC8393443; doi:10.1186/s12872-021-02222-z)
Supplement: Supplementary file 2 — Additional file 2. Table S2: K-codes indicating delivery. [file 12872_2021_2222_MOESM2_ESM.docx]

**SUPPLEMENTAL MATERIAL**

These tables are intended for publication as an online data supplement.

**Supplementary Table S2.** K-codes indicating delivery

| K code |
| --- |
| K891 |
| K892 |
| K893 |
| K894 |
| K894 |
| K895 |
| K8961 |
| K8962 |
| K8963 |
| K8964 |
| K897 |
| K8981 |
| K8982 |
| K900 |
| K9002 |
| K901 |
| K902 |
| K9031 |
| K9032 |
| K9033 |
| K904 |
| K9051 |
| K9051 |
| K9052 |
| K9052 |
| K9061 |
| K9062 |
| K9062 |
| K9063 |
| K907 |
| K908 |
